# The role of spines in anthropogenic seed dispersal on the Galápagos Islands

**DOI:** 10.1002/ece3.6020

**Published:** 2020-01-20

**Authors:** Mae K. A. Johnson, Oscar P. J. Johnson, Reagan A. Johnson, Marc T. J. Johnson

**Affiliations:** ^1^ Clarkson Secondary School Mississauga ON Canada; ^2^ Springfield Public School Mississauga ON Canada; ^3^ St. James Catholic Global Learning Centre Mississauga ON Canada; ^4^ Department of Biology University of Toronto Mississauga Mississauga ON Canada

**Keywords:** anthropogenic disturbance, dispersal ecology, functional trait, gene flow, invasive species

## Abstract

1. Dispersal has important ecological and evolutionary consequences for populations, but understanding the role of specific traits in dispersal can be difficult and requires careful experimentation. Moreover, understanding how humans alter dispersal is an important question, especially on oceanic islands where anthropogenic disturbance through species introductions can dramatically alter native ecosystems.
2. In this study, we investigated the functional role of spines in seed dispersal of the plant caltrop (*Tribulus cistoides* L., Zygophyllaceae) by anthropogenic dispersal agents. We also tested whether humans or wildlife are more important seed dispersers of *T. cistoides* on the Galápagos.
3. *Tribulus cistoides* is found on tropical mainland and oceanic island habitats. The dispersal structure of *T. cistoides* is called a mericarp, and they are typically protected by one pair of upper spines and a second pair of lower spines, but the presence and size of spines varies within and between populations. On the Galápagos, the upper and lower spines protect mericarps from seed predation by Darwin's finches. We tested whether spines play a dual role in dispersal by factorially manipulating the presence/absence of the upper and lower spines to simulate natural variation in mericarp morphology.
4. The upper spines greatly facilitated seed dispersal, whereas the lower spines had no discernible effect on dispersal. The presence of upper spines increased dispersal rate on shoes by pedestrians 23‐fold, on fabrics (e.g., towels) and cars by nearly twofold, and the presence of upper spines increased dispersal distance by cars sixfold. When comparing dispersal rates in habitats with high (roads and foot paths) versus low (arid forest) anthropogenic activity, dispersal rates were demonstrably higher in the habitats with more human activity.
5. These results have important implications for understanding the ecology and evolution of plant dispersal in the Anthropocene. Spines on the fruits of *T. cistoides* play important functional roles in anthropogenic dispersal, whereas native and introduced wildlife plays a minor role in dispersal on inhabited islands of the Galápagos. Our results imply that seed predators and humans are jointly shaping the ecology and evolution of contemporary populations of *T. cistoides* on the Galápagos.

## Graphical abstract

Experimental results showing the effects of upper and lower spines on dispersal of *Tribulus* mericarps by shoes, towels, and cars. In all cases, upper spines facilitated seed dispersal.

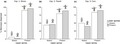

## 1 INTRODUCTION

Dispersal plays an essential biological function for all organisms, with important consequences for the ecological success and evolution of populations (Fenner, 1985; Levin, Muller‐Landau, Nathan, & Chave, 2003). For plants, dispersal away from a parental patch can reduce competition, facilitate establishment in suitable habitats and reduce inbreeding (Howe & Smallwood, 1982; Levin et al., 2003). Many traits have evolved as adaptations to facilitate dispersal and increase fitness, compared to individuals that lack such traits. Examples of dispersal traits in plants include feather‐like plumes and propeller structures for aerial dispersal, fleshy fruits to promote animal consumption and dispersal, buoyancy to allow water dispersal, and hooks and barbs that adhere to fur, feathers, and skin (van der Pijl, 1982). Some plants have no obvious dispersal traits, although the functional significance of a trait in dispersal may be obscured by alternative ecological functions. For example, traits involved in the physical defence against seed predators, could make it difficult to identify additional adaptive roles in dispersal without careful experimentation (Sorensen, 1986). Moreover, the role of specific traits in dispersal may change through time, especially since anthropogenic dispersal has become increasingly prominent for many species (Hulme, 2009).

**Figure 1:**
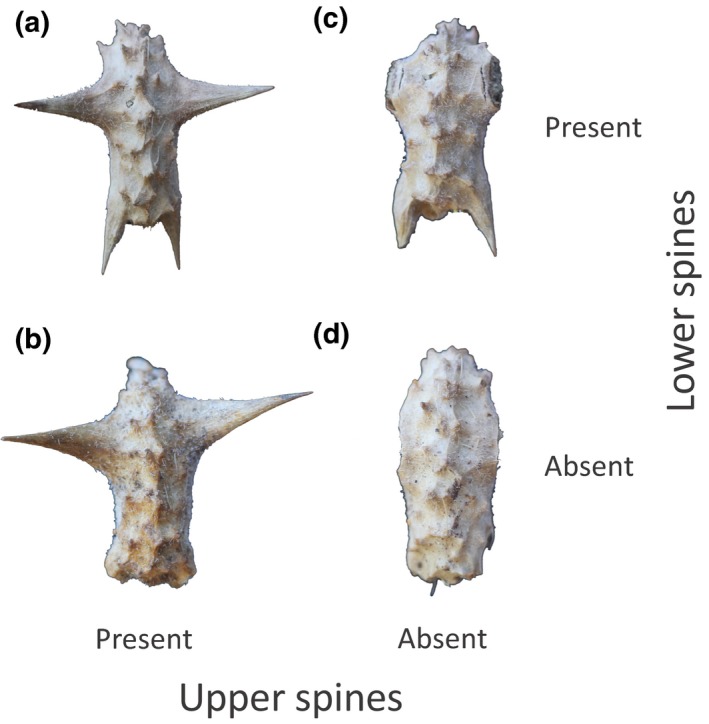
Examples of experimental manipulations of mericarps. All collected mericarps resembled (a), with two upper and two lower spines, which represents the most common morphology on the Galápagos Islands. Mericarps were manipulated using wire cutters to create a 2 × 2 factorial combination of the presence (a & b) or absence (c & d) of upper spines and the presence (a & c) or absence (b & d) of lower spines. See Figure S1 for examples of natural variation in mericarp morphology that spans the variation created by these treatments

Here we examine whether spines that are known to protect seeds against predators also facilitate anthropogenic and nonanthropogenic seed dispersal. As a model, we studied caltrop (*Tribulus cistoides*, Zygophyllaceae) on the Galápagos Islands. This species is well known as an agent of natural selection on beak morphology of Darwin's finches, which drives rapid adaptive evolution of the medium ground finch (*Geospiza fortis*) during extended dry periods (Grant & Grant, 2014). *Tribulus cistoides* (hereafter *Tribulus*) produces segmented fruits called schizocarps that break off into five sections called mericarps, which contain several seeds (Wiggins & Porter, 1971). Mericarps are very hard and difficult to open (Grant, 1981) and are typically protected by four spines, with one pair of spines on the upper dorsal surface and a second pair of spines on the lower dorsal surface of the mericarp (Figure 1a). Although most mericarps have four spines, *Tribulus* vary in spine number on the Galápagos, with some plants producing mericarps with only two spines in either the upper or lower dorsal position, and other plants producing mericarps that completely lack spines (Carvajal‐Endara et al., 2020; Grant, 1981; Figure S1). Moreover, although all *Tribulus* spp. have mericarp spines, the closest relatives of *Tribulus* (*Kallstroemia*, *Tribulopis*, *Kelleronia*) (Sheahan & Chase, 2000), either lack spines or have small, poorly developed spines or rounded protuberances, instead of long sharp spines (Barker, 1998; Sheahan, 2007). This suggests that the spines of *Tribulus* are a derived character that likely evolved in response to natural selection. However, there continues to be variation in the presence, number and size of spines within *Tribulus* populations on the Galápagos, leading to the question of what role do these spines play in defence or dispersal?

**Figure 2:**
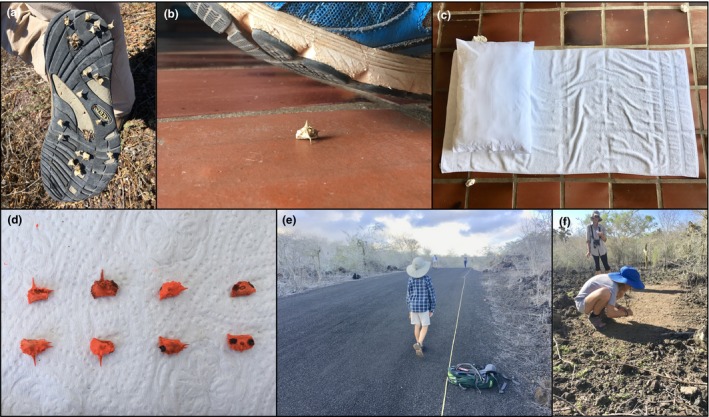
Photographs illustrating important natural history observations and experimental methodology. (a) *Tribulus* frequently adheres to shoes and tires (not depicted). (b) Example of how *Tribulus* dispersal by shoes was tested in Experiment 1. (c) Illustration of how a towel was placed overtop of mericarps and then evenly compressed over its entire surface with a pillow in Experiment 2 before lifting the towel. (d) Examples of spray‐painted mericarps used in Experiment 3, with the four treatments shown from left to right in the following order: all spines intact, lower spines removed, upper spines removed, and all spines removed. (e) Road habitat from Experiment 3 where mericarps were placed for 52 hr followed by measuring the dispersal distance using a measuring tape. (f) Example of natural habitat used in Experiment 4, with rocks cleared prior to placing mericarps to facilitate mericarp recovery after 48 hr

A combination of recent experiments and natural history observations suggest that seed predators and dispersal may impose selection on spines. A recent study showed that spines act as an antipredator defence against three Darwin's finch species (*G. fortis*, *G. magnirostris*, *G. conirostris*), whereby upper and lower spines reduce seed predation (Carvajal‐Endara et al., 2020). However, this selection varied in space and time depending on climatic variation and the presence of large‐beaked finch species (*G. magnirostris* and *G. conirostris*). Moreover, variation in mericarp morphology was not always consistent with the direction predicted by selection from seed predators, suggesting that variation in spines is explained by additional factors other than seed predation. It has been hypothesized that spines can play a dual role in dispersal (Sorensen, 1986). For example, the spines of *Tribulus* mericarps have been observed to adhere to the soles of shoes (Figure 2a), tires, animal feet, and they are hypothesized to adhere to feathers and fur (Carlquist, 1967; van der Pijl, 1982; Sheahan, 2007). Thus, spines may facilitate defence against predators and simultaneously aid seed dispersal, and these factors may jointly affect the evolution of spines.

To understand the role of spines in dispersal and the importance of humans as dispersal agents on the Galápagos Islands, we asked four research questions. First, do spines affect seed dispersal of *Tribulus*? If spines facilitate dispersal, then we expect dispersal to be greater for mericarps that have spines, as opposed to mericarps that lack spines. Second, does the importance of spines in dispersal vary with their position on the fruit? Although *Tribulus* typically has four spines distributed as an upper and lower set, both of which reduce seed predation by Darwin's finches, perhaps only a subset of spines are involved in dispersal. The common observation of *Tribulus* being dispersed on tires and shoes suggests that at least in some locations, humans play a role in dispersal. Thus, we asked a third question: On what types of materials do humans facilitate dispersal of mericarps? We specifically tested the role of dispersal by shoes, towels, and cars, since *Tribulus* is commonly found along foot paths, beaches, and roadways. Finally, what are the relative roles of anthropogenic versus nonanthropogenic dispersal of *Tribulus* seeds on the Galápagos? If wildlife are the most important dispersers, then we expect higher dispersal rates in natural areas where humans seldom visit.

## 2 METHODS

### 2.1 Study system

*Tribulus cistoides* L (Zygophyllaceae) is a perennial herbaceous plant that grows in tropical regions throughout the world. Its native status in the Galápagos Islands is unclear, with some describing the species as native (Svenson, 1930), speculating that it was carried to the archipelago by birds (Carlquist, 1967; Grant, Smith, Grant, Abbott, & Abbott, 1975), whereas others suggest it was introduced more recently by sailors (Grant & Grant, 2014; Porter, 1976; Wiggins & Porter, 1971). It has been present on the Galápagos since at least before 1835, when Darwin collected the first specimens from two islands (Hooker, 1847). On the Galápagos, it grows only in the arid zone where it is found along beaches and rocky shorelines, in bare soil and gravel at the edges of roads, in lava fields and up to the base of the transitional zone. It has a prostrate growth form, with long branches that run along the soil surface, extending out from a single root stock. Plants can flower at any time, but they flower the most during the wet season. As the fruits mature and dry, five individual indehiscent mericarps, which each contain one to seven seeds, break off and fall beneath the plant. This is when long‐distance dispersal is possible, and our experiments focused on dispersal at this stage.

### 2.2 Experimental design

All of our experiments were conducted on the island of Santa Cruz, Galápagos Islands, Ecuador. We conducted three separate experiments to determine the function of spines in dispersal by humans. We conducted a fourth experiment to test the relative roles of dispersal by wildlife and humans. All mericarps were collected from a single large population adjacent to the main port in Puerto Ayoro. We used only mericarps that were undamaged and had four spines (two upper and two lower spines). Mericarps were always placed so that they rested naturally on the ground, their ventral surface facing downward and their upper spines pointing upward, almost perpendicular to the ground. This was done because a survey of 285 naturally occurring mericarps revealed that 70% were in this orientation. We removed spines using wire cutters to simulate mericarps that were lacking upper spines, lower spines or lacking all spines (Figure 1, Figure S1). We describe each of the experiments below.

#### 2.2.1 Experiment 1: Dispersal by shoes

Mericarps are frequently found on the soles of shoes, so we conducted an experiment to test the role spines play in dispersal on the shoes of pedestrians (Figure 2). Two hundred intact mericarps were used and the presence and absence of upper and lower spines were manipulated in a fully factorial manner with balanced replication: 50 intact mericarps (control), 50 mericarps with upper spines absent, 50 mericarps with lower spines absent, 50 without upper or lower spines (Figure 1). Two individuals wearing shoes with rubber soles walked over each mericarp once (i.e., two trials per mericarp), and each individual continued walking for five steps (>2 m) (Figure 2b). The mericarp was recorded as “dispersed” if a mericarp became lodged in a shoe sole and did not fall before five steps were taken in either trial.

#### 2.2.2 Experiment 2: Dispersal by towels

*Tribulus* frequently grows on or near beaches that are used recreationally, so we tested if spines facilitate dispersal on fabrics such as beach towels. We used a total of 400 mericarps that were factorially manipulated for the presence/absence of upper and lower spines as described for experiment 1, again with balanced replication across all four treatment combinations (100 replicates per treatment). Mericarps were placed on a hard floor, and a towel was spread flat overtop of the seeds, which was then patted down gently and evenly across the entire surface using a pillow to simulate the weight of a person laying on a beach (Figure 2c). The towel was then gently lifted, turned over 180°, and each mericarp was scored as to whether or not it adhered to the towel (dispersed) or remained on the floor (not dispersed); mericarps that fell to the ground while lifting and turning the towel were counted as not dispersed. We conducted four trials, with 100 mericarps per trial, with equal replication of all treatments in each trial.

#### 2.2.3 Experiment 3: Dispersal by cars

*Tribulus* commonly occurs along roadsides, providing an opportunity for cars to disperse mericarps that become lodged in tires. We examined this possibility by placing mericarps on a roadway for 52 hr and then scoring whether mericarps were dispersed (moved >1 m from their original location) or not dispersed (moved <1 m from the original location). We also recorded dispersal distance to the nearest meter with the maximum distance set to 30 m. The experiment was conducted on the paved road leading to El Garrapatero beach, with mericarps placed ca. 2 km from the end of the road. Taxis frequently pass (e.g., 17 per hr during experimental setup) to transport people to and from the beach. We placed 200 mericarps in a line running across the road, so that mericarps could be picked up by cars traveling in either direction. Each mericarp was spray‐painted fluorescent orange so that they could be easily found following the experiment, but mericarps were small enough that they were inconspicuous to drivers. Importantly, the paint did not change mericarp morphology (Figure 2d). Mericarps only moved when there was direct contact between the mericarp and wheels; they are large enough that they do not blow around when vehicles pass. The presence of upper and lower spines was factorially manipulated as described above, with each treatment coded using dots using a black Sharpie Permanent Marker (Sanford, LP), which allowed us to ascertain the treatment applied to a mericarp even when spines were broken by tires (Figure 2d). The treatments were placed on the road in an alternating pattern, so that all four treatments were equally represented across the width of the road. After 52 hr of exposure to cars, we carefully searched both sides of the road and shoulder for a distance of 50 m in both directions from the starting point (Figure 2e). Mericarps were readily spotted and recovered up to 30 m away from the start point, with only three mericarps found >30 m away. Mericarps that were not recovered were likely lodged in vehicle tires and marked as dispersed 30 m.

#### 2.2.4 Experiment 4: Anthropogenic versus nonanthropogenic dispersal

We sought to understand the relative roles of humans versus wildlife in dispersing *Tribulus* mericarps in contemporary communities. This was done by placing 50 mericarps in each of six separate locations on Santa Cruz island, which represented three habitat types. One habitat was roads primarily used by cars or bicycles, which included the road to El Garrapatero and the road leading into the Charles Darwin Research Station (CDRS). The second habitat was pedestrian walkways where there was heavy foot traffic but no cars; we used a brick‐laid footpath in Puerto Ayoro, and a gravel path frequented by visitors to CDRS. The third habitat was natural areas where people rarely travel, which included an arid lava boulder area with patches of bare ground among cactus near El Garrapatero (ca. 100 m from the road) (Figure 2e), and an arid open forest ca. 500 m east of CDRS. In each location there were naturally occurring *Tribulus* within 100 m and often immediately adjacent to the site. The first two habitats receive heavy anthropogenic activity, as well as activity from native birds such as Darwin's finches and Galápagos Mockingbirds. The roadway to CDRS is also frequented by marine iguanas. The natural locations receive almost no anthropogenic activity, but are visited by native (e.g., Giant Tortoise) and introduced wildlife (e.g., goats), as well as birds. Mericarps were spray‐painted white or black to increase their contrast on the background substrate, enabling us to distinguish them from naturally occurring *Tribulus*. However, since these colors are common colors of natural substrates on the islands, they were not easily noticed by pedestrians or wildlife. Mericarps were spaced in discrete rows over an area of 2–5 m depending on the dimensions and makeup of the habitat. In the natural areas, rocks and pebbles were brushed away using a broom to create a smooth, flat surface (Figure 2f). We returned to each site after 48 hr, collected all mericarps and determined how many mericarps had been dispersed >1 m or were not dispersed.

### 2.3 Statistical analyses

Generalized linear models were implemented in R 3.4.0 for all analyses to test how the presence/absence of upper and lower spines affected dispersal by shoes, towels, and cars (R Core Team, 2013). We treated dispersal as a binary response variable with 1 and 0 corresponding to mericarps that were either dispersed or not dispersed, respectively. Dispersal was fit using the function *glm* to the following model: Dispersal = Upper spines + Lower spines + Upper spines x Lower spines, with the distribution stipulated as *family = binomial*. The statistical significance of each effect was assessed using *P*‐values from analysis of deviance (type II error) implemented using the *car* package (Fox & Weisberg, 2011). Experiment 1 included mericarp mass as a covariate, but this effect was nonsignificant (*p* = .26) and removed from the final model. We also modeled dispersal distance of mericarps by cars in experiment 3, which was fit to a negative binomial distribution, which provided a better fit than Poisson, normal or zero‐inflated distributions of these models. For experiment 4, we again used *glm* fit to a binomial distribution to test how habitat type (road, footpath, natural area) affected dispersal. We further classified these habitats as low (natural area) versus high (road and footpath) anthropogenic activity, to determine the relative roles of humans and wildlife in dispersal.

## 3 RESULTS

### 3.1 Experiment 1: Dispersal by shoes

**Figure 3:**
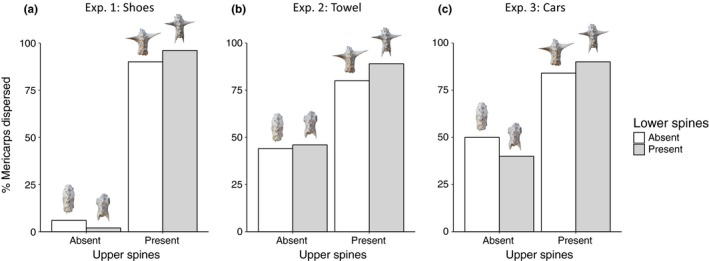
Experimental results showing the effects of upper and lower spines on dispersal of *Tribulus* mericarps by (a) shoes, (b) towels, and (c) cars. The presence/absence of spines was manipulated using wire cutters; representative mericarps with treatment manipulations are shown above each bar. Dispersal was treated as a binomial response variable, with the bars showing the total % of mericarps that were dispersed

*Tribulus* mericarps were readily dispersed by shoes, but only when the upper spines were intact. The presence of upper spines increased mericarp dispersal (χ^2^ = 94.36, *p* < 0.001), with 93% of mericarps being dispersed by shoes when upper spines were intact versus only 4% of mericarps being dispersed when upper spines were absent (Figure 3a). The presence of lower spines had no clear effect on dispersal (χ^2^ = 0.10, *p* = .76), with dispersal being similar in the presence and absence of lower spines (Figure 3a). The presence of upper and lower spines did not interact to affect dispersal (χ^2^ = 2.42, *p* = .76).

### 3.2 Experiment 2: Dispersal by towels

Across all treatments, 65% of *Tribulus* mericarps adhered to towels, with the presence of upper spines nearly doubling dispersal rate (Figure 3b). This effect of upper spines was highly significant (χ^2^ = 94.39, *p* < 0.001), whereas the presence of lower spines (χ^2^ = 0.014, *p* = 0.90) and the interaction between upper and lower spines (χ^2^ = 0.035, *p* = 0.85) had no discernible effects on dispersal.

### 3.3 Experiment 3: Dispersal by cars

Upper spines facilitated the probability and distance of dispersal by cars. Upper spines increased mericarp dispersal from 45% (upper spines absent) to 87% (upper spines present) (Figure 3c), an effect that was highly significant (χ^2^ = 41.53, *p* < 0.001). Upper spines also led to a sixfold increase in the average distance of dispersal, from 2.1 m (upper spines absent) to 12.6 m (upper spines present) (χ^2^ = 58.29, *p* < 0.01). As with experiments 1 and 2, lower spines and the interaction between upper and lower spines had no discernible effects on mericarp dispersal (all *p* > .15; Figure 3c).

### 3.4 Experiment 4: Anthropogenic versus nonanthropogenic dispersal

**Figure 4:**
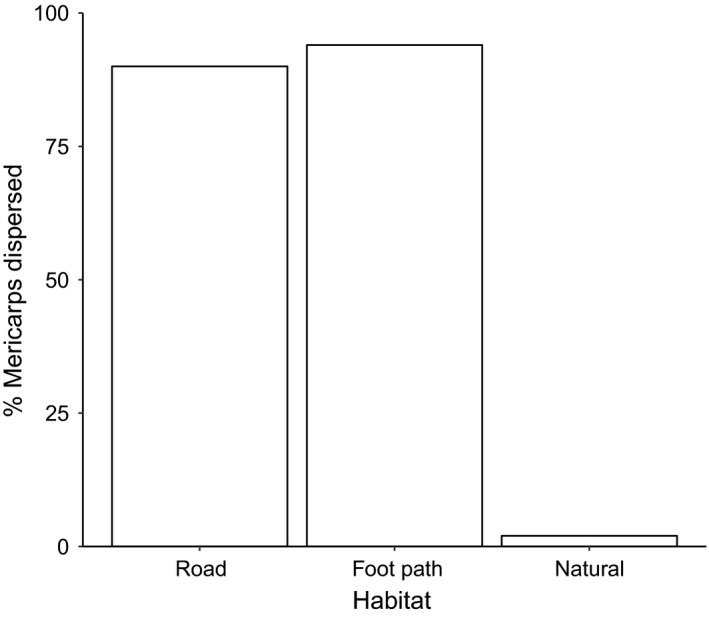
Mericarp dispersal in habitats with high (roads and foot paths) and low (natural) anthropogenic activity. Dispersal was treated as a binomial response variable with the height of the bars showing the total % of mericarps that were dispersed. Cars and bikes were the primary dispersal agents on roads; pedestrians were the principal dispersal agent on foot paths, and native and introduced wildlife was the main source of dispersal in natural areas

The rate of dispersal was demonstrably higher in habitats with high anthropogenic activity versus low anthropogenic activity. The rate of dispersal over 48 hr was ≥90% on both foot paths and roads, and just 2% in areas primarily visited by wildlife (Figure 4). This large difference was reflected by a highly significant difference in dispersal rate among the three habitats (χ^2^ = 268.42, *p* < 0.01) and when habitats were categorized dichotomously as high (foot paths and roads) versus low anthropogenic activity (natural habitat) (χ^2^ = 267.32, *p* < 0.01).

## 4 DISCUSSION

Our results lead to several conclusions about the role of spines and humans in the dispersal of *Tribulus* on the Galápagos Islands. First, dispersal of *Tribulus* seeds is facilitated by the presence of upper spines, whereas lower spines have no clear effect on dispersal (Questions 1 and 2). Second, it is clear that humans can disperse *Tribulus* propagules by multiple means, including on shoes, tires, and on fabrics such as towels (Question 3). Finally, our results suggest that humans are the primary agents of dispersal of *T. cistoides* on inhabited islands of the Galápagos, whereas native and introduced wildlife appears to be of lesser importance in contemporary communities (Question 4). We discuss the significance of these conclusions for understanding the roles of spines in defence and dispersal, and specifically, how humans affect the ecology and evolution of plants on the Galápagos Islands.

### 4.1 Role of spines in dispersal

Spines are typically viewed as morphological defences against predators and herbivores (Meredith, Tindall, Hemmings, & Moles, 2019; Tindall, Thomson, Laffan, & Moles, 2016). Spines are frequently found on many plant parts, including leaves, stems, flowers, and fruits (Tindall et al., 2016). While the primary function of spines on most plant parts is undoubtedly related to defence, the function of spines on fruits is less clear because they may play both a role in defence against seed predators and dispersal of seeds away from the parent plant (Sorensen, 1986). For example, many plants have hooks, barbs, and spines that adhere to animal fur, bird feathers, and feet (Sorensen, 1986). In the case of *T. cistoides*, a recent study showed that spines reduce seed predation by Darwin's finches on the Galápagos Islands (Carvajal‐Endara et al., 2020). Longer upper spines and the presence of lower spines reduced seed predation, and this was especially important on islands with large‐beaked finches and following dry years when seed sources were diminished. This result supported earlier evidence of the role of spines in defence against *G. fortis* on Isla Daphne Major in the Galápagos (Grant, 1981). Despite this selection, islands with the largest beaked finch species did not have the longest spines as one would expect based on empirical measures of selection, although the presence of lower spines was more common on these islands. These results suggest that seed predation on its own cannot explain the evolution and distribution of mericarp morphology on the Galápagos Islands.

Our results clearly show that spines play a role in dispersal of *Tribulus* mericarps on the Galápagos. However, only upper spines facilitated seed dispersal, whereas lower spines had no clear effect on dispersal. The different functions of upper and lower spines are likely attributed to the fact that upper spines are typically oriented so they are pointing upwards, almost perpendicular to the ground, whereas the lower spines are often shorter and pointing outward in an orientation that likely makes it more difficult for them to adhere to surfaces (Figure 2, Figure S1). These findings suggest that both seed predation and dispersal likely affect adaptive evolution of upper spines, whereas adaptive evolution of lower spines is solely affected by seed predators on the Galápagos (Carvajal‐Endara et al., 2020). It has already been demonstrated that the strength and direction of selection by seed predators varies across islands as a function of the finch community, and in response to variation in annual precipitation (Carvajal‐Endara et al., 2020). We do not know how seed dispersal varies among islands, but given that most islands are uninhabited by humans, and in light of the prominent role of anthropogenic seed dispersal on the Galápagos (see below), it is likely that selection on upper spines imposed by dispersal agents varies tremendously. The existing variation in mericarp morphology on the Galápagos Islands, which includes variation in the size and presence/absence of upper and lower spines (Figure S1), likely reflects a combination of selection by seed predators and dispersers. For example, it is possible that dispersal and defence are maladaptive on some islands, especially if there are few ground finches and if dispersal leads to movement to unfavorable habitats. Such a scenario could lead to selection for the loss of spines. These possibilities are speculative and do not account for genetic drift, founder events, and gene flow, which will also affect evolution of *Tribulus*. A combination of experiments that manipulate selection by seed predators and dispersers, coupled with population genomic analyses across multiple islands could help to resolve the role that seed predators, dispersal agents, and nonadaptive processes play in the evolution of spines and dispersal history of plants.

### 4.2 Anthropogenic dispersal

Our results suggest that humans are the primary agents of dispersal for *Tribulus* on the Galápagos Islands. Experiment 4 showed that humans are capable of dispersing almost all of the mericarps, even in a short period of time. By contrast, mericarp dispersal was very low in natural habitats. These results, combined with the observation that *Tribulus* is primarily found along roadways, footpaths, and beaches on the Galápagos Islands, implicate humans as the primary dispersers, at least on inhabited islands and islands visited by tourists. This does not rule out birds and other animals from dispersing *Tribulus*, since *T. cistoides* can also be found growing in close association with seabird colonies (D. Reyes, personal communications). Unfortunately, our permits did not allow us to study seed dispersal in seabird colonies, and the role of seabirds in seed dispersal requires further experimentation, because most conclusions about the role of seabirds in dispersal of *T. cistoides* on oceanic islands are based on speculation and fortuitous natural history observations (Carlquist, 1967; Porter, 1976). Nevertheless, given the large difference in rates of dispersal in areas with high versus low anthropogenic activity, it is clear that humans are likely to be the most prominent dispersal agents in contemporary communities.

The relative roles of humans and wildlife in dispersing *Tribulus* also have implications for understanding how this species originated on the Galápagos Islands. Some have speculated that *T. cistoides* arrived to the archipelago by natural means and has been on the Galápagos for a long period of time (Carlquist, 1967). Others have speculated that it was introduced after humans first visited the islands in 1535 (Grant, 1981; Porter, 1967), but before Charles Darwin collected the first specimens on the archipelago in 1835 (Hooker, 1847). This is an important question because it is the consumption of *Tribulus* which is credited with driving the rapid evolution of beak size in *G. fortis* (Grant & Grant, 2002, 2014), and potentially adaptive divergence and speciation of different Darwin's finch species as they have hybridized and evolved to specialize on different food resources due to character displacement (Boag & Grant, 1984; Grant & Grant, 2006; Lamichhaney et al., 2016). If humans brought *T. cistoides* to the Galápagos Islands, then one of evolutionary biology's best examples of evolution in nature is driven by an introduced species, which would further inform our understanding of how humans are fundamentally changing the ecology and evolution of life on Earth (Otto, 2018). Our results suggest that humans likely had the capacity to disperse *Tribulus* to the Galápagos. Indeed, *T. cistoides* is found on many oceanic islands in the tropics throughout the world. However, our observations do not allow for any definitive conclusions about the origin of *Tribulus* on the Galápagos Islands. Phylogeographic and demographic analyses based on genomic data are required to convincingly test the timing and source of populations. Such analyses would provide important insight into the historical and contemporary ecology and evolution of this plant.

### 4.3 Limitations of the study

There are two important limitations to consider when interpreting our results. First, our results may have been biased in favor of detecting high dispersal rates. Mericarps were always oriented with the upper spines pointed upwards, because surveys showed that 70% of mericarps occur in this orientation within natural populations. The remaining 30% of mericarps have their upper spines pointing downwards in natural populations. Our results reflect the most common scenario in which mericarps are most likely to be dispersed. If we had manipulated the mericarps to have the naturally occurring ratio of spines facing upwards and downwards, the overall dispersal rates would have likely been lower, but we expect the results would be qualitatively identical.

The second limitation is that although we considered dispersal from multiple habitats, we did not investigate the fate of dispersed seeds and whether they reach sites suitable for germination. It is likely that many seeds are dispersed to unsuitable sites, and this frequency may vary among habitats. For example, dispersal along paths may lead to a high rate of successful dispersal events in which propagules reach sites safe for germination, whereas dispersal along paved roads may be disproportionately associated with dispersal to unsuitable habitats (e.g., asphalt). However, given the high abundance of *Tribulus* along roadsides, unpaved roads, and parking lots, roads likely represent an important conduit of successful dispersal. Controlled dispersal experiments coupled with genotyping of parents and progeny would help to resolve the variation in the success of dispersal events.

### 4.4 Conclusions

Our experiments have general implications for understanding seed dispersal in the Anthropocene. First, our experiments combined with the results of Carvajal‐Endara et al. (2020) clearly show that spines on fruits can play important functional roles in both dispersal and predation. This implies that in such cases, the evolution of spines will depend on simultaneous selection by antagonistic interactions with seed predators and commensal interactions involving happenstance dispersal. Our results also reinforce the view that humans are having a dramatic impact on the movement of organisms (Hulme, 2009), including in remote regions like the Galápagos Islands (Porter, 1976), where conservation efforts are underway to preserve the natural environments of the archipelago while capitalizing on the economic benefits of ecotourism. This prominent anthropogenic dispersal is well known to alter the ecology of species and communities, but it is also increasingly appreciated that such anthropogenic pressures can drive rapid evolution (Johnson & Munshi‐South, 2017; Otto, 2018).

## CONFLICT OF INTEREST

The authors declare no conflicts of interest.

## AUTHORS’ CONTRIBUTIONS

MKAJ, OPJJ, MTJJ interpreted the results and conceived and designed the experiments. All authors constructed the experiments and collected the data. MTJJ conducted the analyses and wrote the first draft of the paper, and all authors edited subsequent drafts.

## Data Availability

Data and R scripts for statistical analyses and figures are available on Data Dryad (https://doi.org/10.5061/dryad.t1g1jwsz8).
