# Supplementary material for: The role of spines in anthropogenic seed dispersal on the Galápagos Islands
Source: Ecol Evol. 2020 Jan 20;10(3):1639–47. doi: 10.1002/ece3.6020 (PMC7029089; doi:10.1002/ece3.6020)
Supplement: Supplementary file 1 [file ECE3-10-1639-s001.pdf]

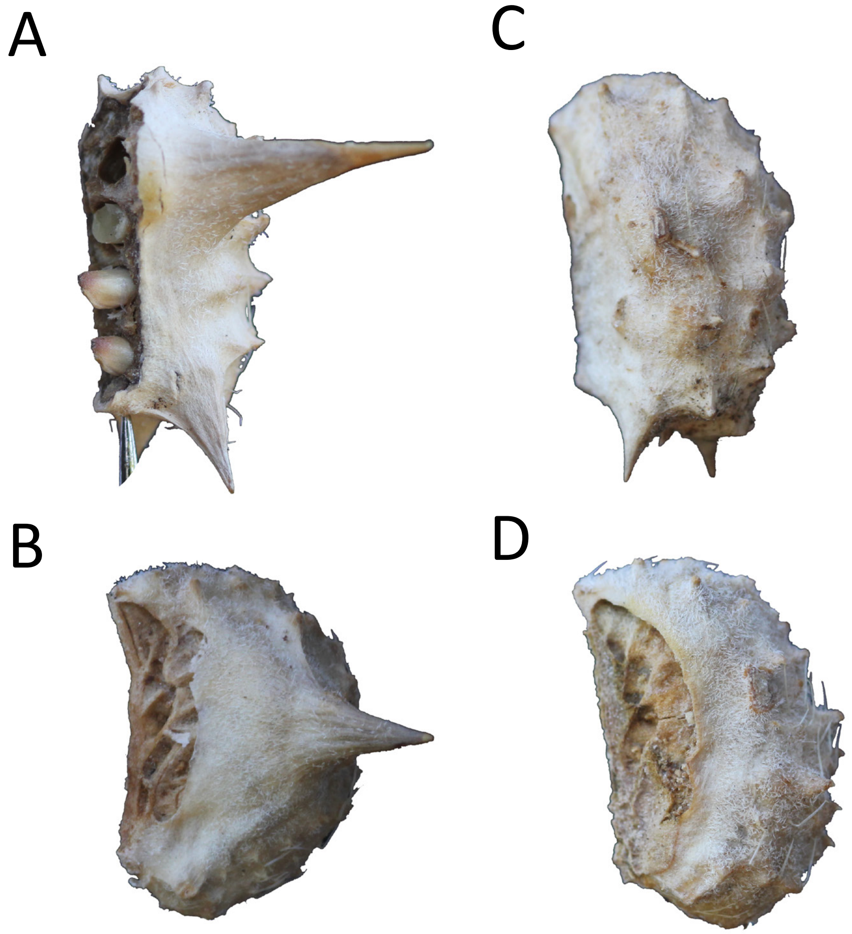

FIGURE S1 Natural variation in mericarp morphology on the Galápagos Islands. (A) Most plants produce mericarps that have four spines, distributed as two large upper spines and two smaller lower spines; ventral surface cut away to show seeds contained within the mericarp (B) Plants that have two prominent upper spines and lack lower spines are also common, whereas plants (C) lacking upper spines but with lower spines, or (D) lacking all spines, occur but are infrequent.
